# Oculopharyngeal Muscular Dystrophy: A Case Report From Puerto Rico

**DOI:** 10.7759/cureus.65766

**Published:** 2024-07-30

**Authors:** Julian A Menendez Sepulveda, Natalio Izquierdo

**Affiliations:** 1 Medical School, University of Puerto Rico, Medical Sciences Campus, San Juan, PRI; 2 Department of Surgery, University of Puerto Rico, Medical Sciences Campus, San Juan, PRI

**Keywords:** blepharoptosis, general ophthalmology, trinucleotide repeat defect, genetic muscle disorders, oculopharyngeal muscular dystrophy

## Abstract

Oculopharyngeal muscular dystrophy (OPMD) is a late-onset inherited skeletal myopathy. The diagnosis is based on a clinical presentation of blepharoptosis, dysphagia, and a positive family history of the disease in patients past 40 years of age. A 57-year-old male patient presented with ptosis without lid crease, adult-onset dysphagia, and bilateral pseudophakia. The patient underwent ptosis repair of upper eyelids via frontalis slings with silicone rods. His mother was subsequently found to have ptosis, dry eyes, and anorexia due to dysphagia, thus suggesting a probable family history. Based on the comprehensive ophthalmic evaluation, and based on his ptosis, dysphagia, and family history, the patient was diagnosed with OPMD.

## Introduction

Oculopharyngeal muscular dystrophy (OPMD) is a late-onset inherited skeletal myopathy presenting with blepharoptosis, dysphagia, and proximal muscle weakness [1]. Diagnosis is based on a clinical presentation of blepharoptosis, dysphagia, and a positive family history of the disease in a patient older than 40 years of age [1,2]. In atypical presentations, histological analyses and electromyographic studies may be conducted to exclude other potential diagnoses [1]. Life expectancy is unaffected, albeit quality of life is severely impacted in the late stages of the disease [2,3]. These patients should be referred for confirmatory genetic analysis with polymerase chain reaction (PCR) testing which is the gold standard for diagnosis [4,5].

The disorder is caused by trinucleotide expansions of the wild-type (*GCN*)10 repeats in the polyadenosine binding protein nuclear 1 (*PABPN1*) gene [1,6]. OPMD manifests initially between the fifth and sixth decades with progressive blepharoptosis, dysphagia, and lower limb weakness which ascends to upper limbs and other voluntary muscles [5]. Later stages of the disease have variable symptomatology and heterogenous phenotype depending on the severity and progression of the myopathy but may include tongue atrophy and weakness, dysphonia, limitation of upper gaze, partial horizontal ophthalmoplegia, facial muscle weakness, loss of tendon reflexes without clear polyneuropathy, and reduced forced expiratory volume in one second (FEV1) [5,7].

Current treatment for OPMD is primarily surgical. For blepharoptosis, two procedures have been described with acceptable results, resection of the levator palpebrae superioris (LPS) aponeurosis, and frontalis suspension of the upper eyelid [1,4,5]. For dysphagia, botulin toxin, cricopharyngeal myotomy, and autologous satellite cell transplants may be performed; however, the latter has recently been disputed due to evidence that the telomeres of satellite cells and myoblasts, and, therefore, replicative and proliferative abilities are significantly disrupted in muscle dystrophies such as OPMD [1,4,5,8]. As the exact cellular pathogenesis of OPMD is yet to be elucidated, many pharmacological treatments are in animal model studies based on the treatment of other muscular dystrophies or trinucleotide repeat diseases, and some include the use of chaperones, monoclonal antibodies, doxycycline, and muscle hypertrophy induction. [1,4]

Inheritance depends on the number of repeats; 12-18 repeats manifest as autosomal dominant with complete penetrance, while 11 repeats were previously thought to only present as autosomal recessive, but a new, possibly de novo, mutation has been described as autosomal dominant [1-7,9]. While no genetic anticipation has been evidenced, the age of diagnosis and disease severity have been correlated with the length of trinucleotide expansion; a longer expansion entails a younger age at diagnosis and a more severe phenotype [9].

## Case presentation

A 57-year-old male patient presented with intellectual disability, ptosis without lid crease, and extended neck position. He also had adult-onset dysphagia, leading to weight loss.

The patient underwent a comprehensive ophthalmic evaluation. His best-corrected visual acuity (BCVA) was 20/50 and 20/40 in the right and left eye, respectively. Refraction was -0.50 +0.75 x 90˚ in both eyes. He had bilateral pseudophakia and normal indirect ophthalmoscopy findings. Since the patient had ptosis, he was referred to an oculoplastic surgeon for ptosis repair of upper eyelids via frontalis slings with silicone rods. His ptosis improved.

**Figure 1:**
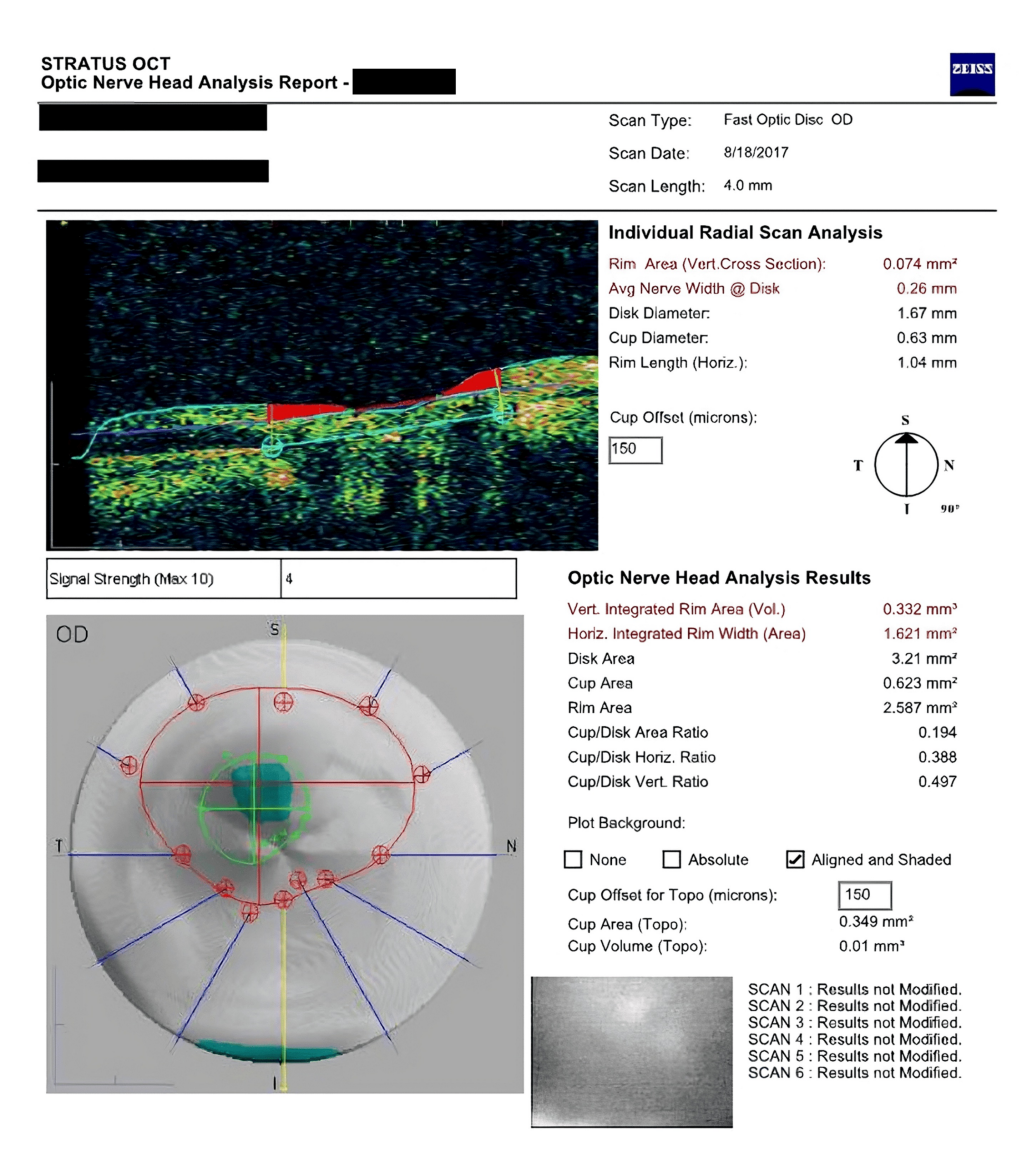
Patient's right eye OCT showing disk-to-cup ratio of 0.2 (Zeiss Stratus*) OCT: optical coherence tomography *Carl Zeiss Meditec AG, Jena, Germany

**Figure 2:**
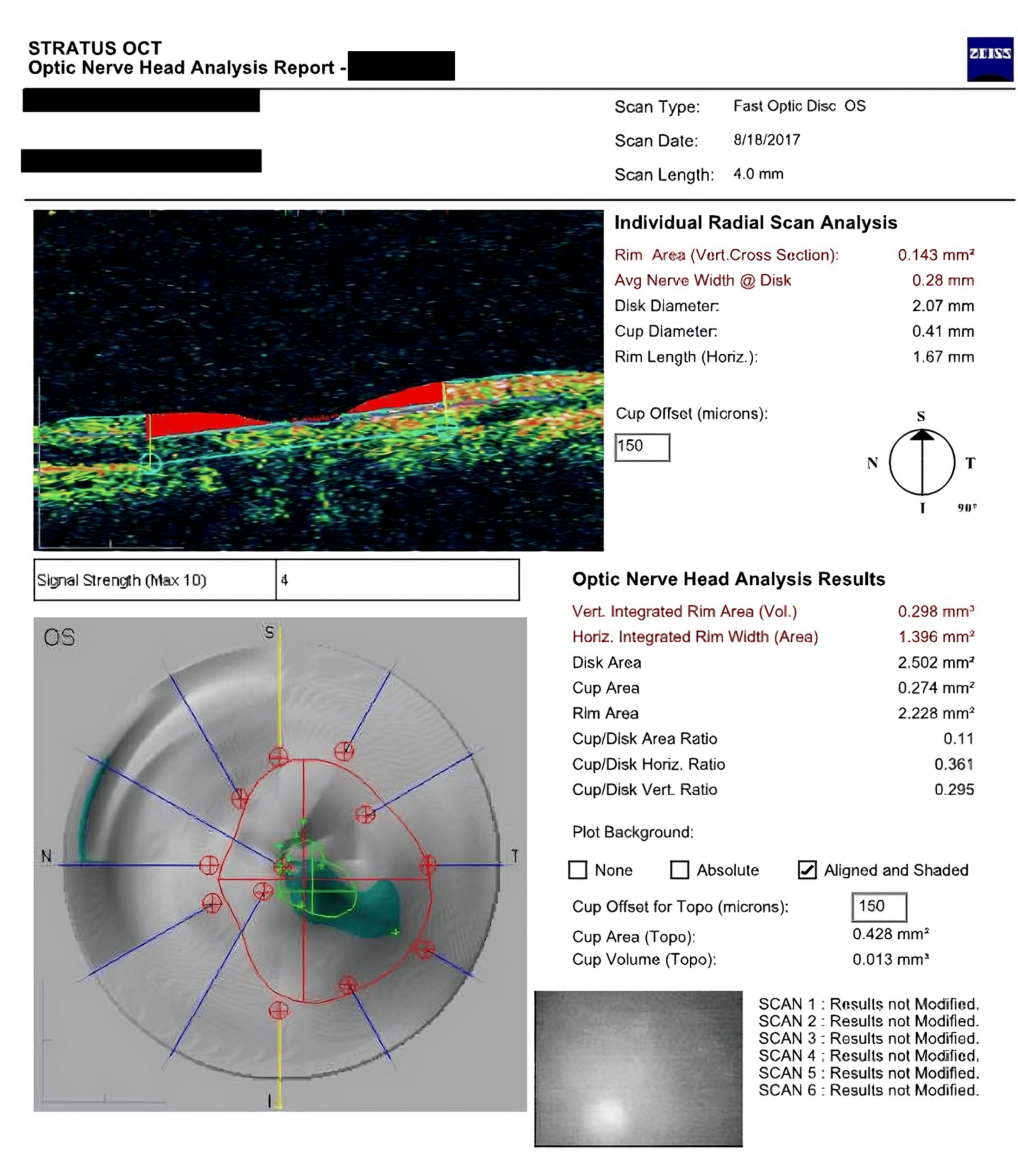
Patient's left eye OCT showing disk-to-cup ratio of 0.1 (Zeiss Stratus*) OCT: optical coherence tomography *Carl Zeiss Meditec AG, Jena, Germany

**Figure 3:**
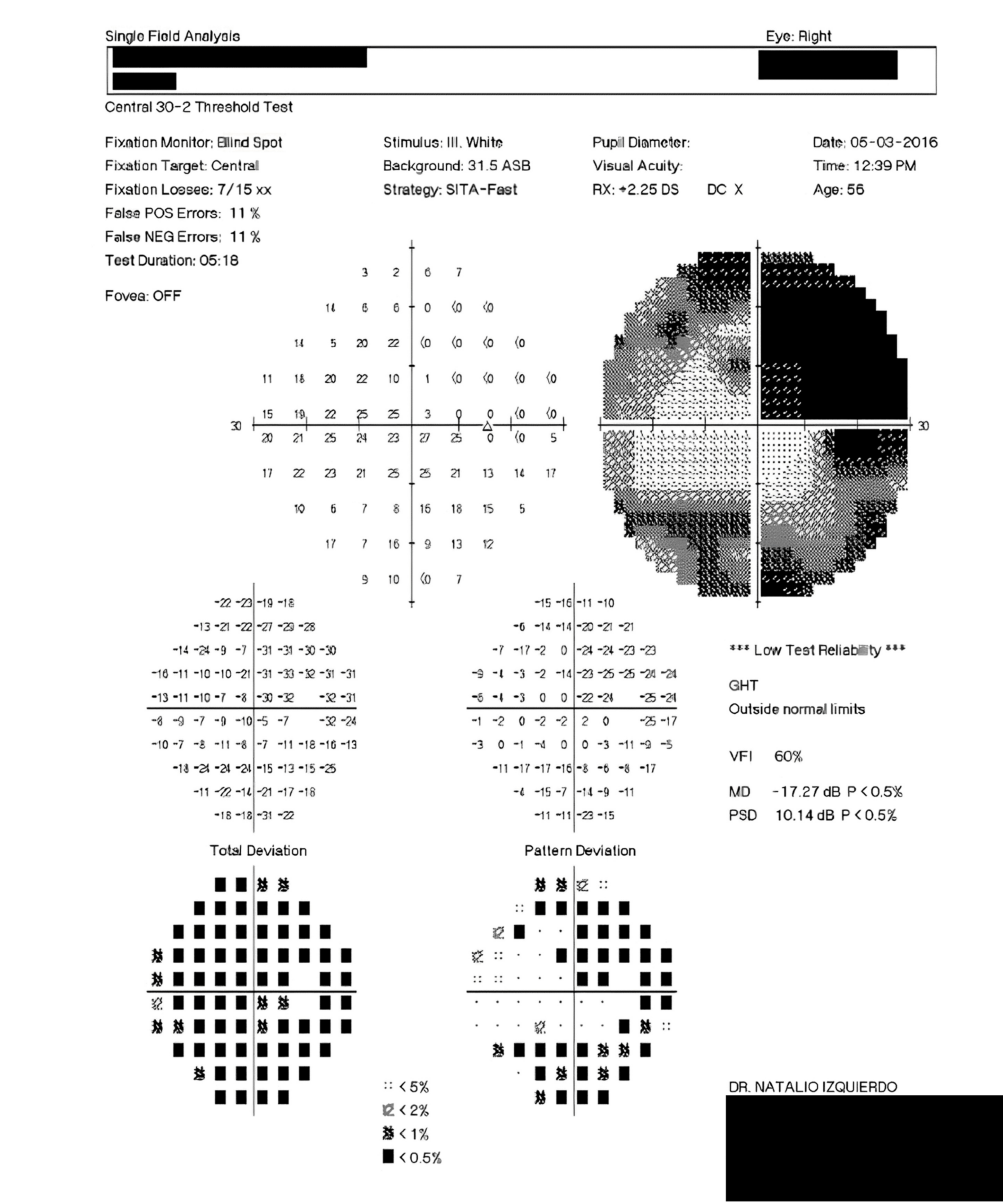
Patient's right eye Humphrey visual field test showing mean deviation of -17.27 dB (30-2*) *Carl Zeiss Meditec AG, Jena, Germany GHT: glaucoma hemifield test; VFI: visual field index; MD: mean deviation; PSD: pattern standard deviation

**Figure 4:**
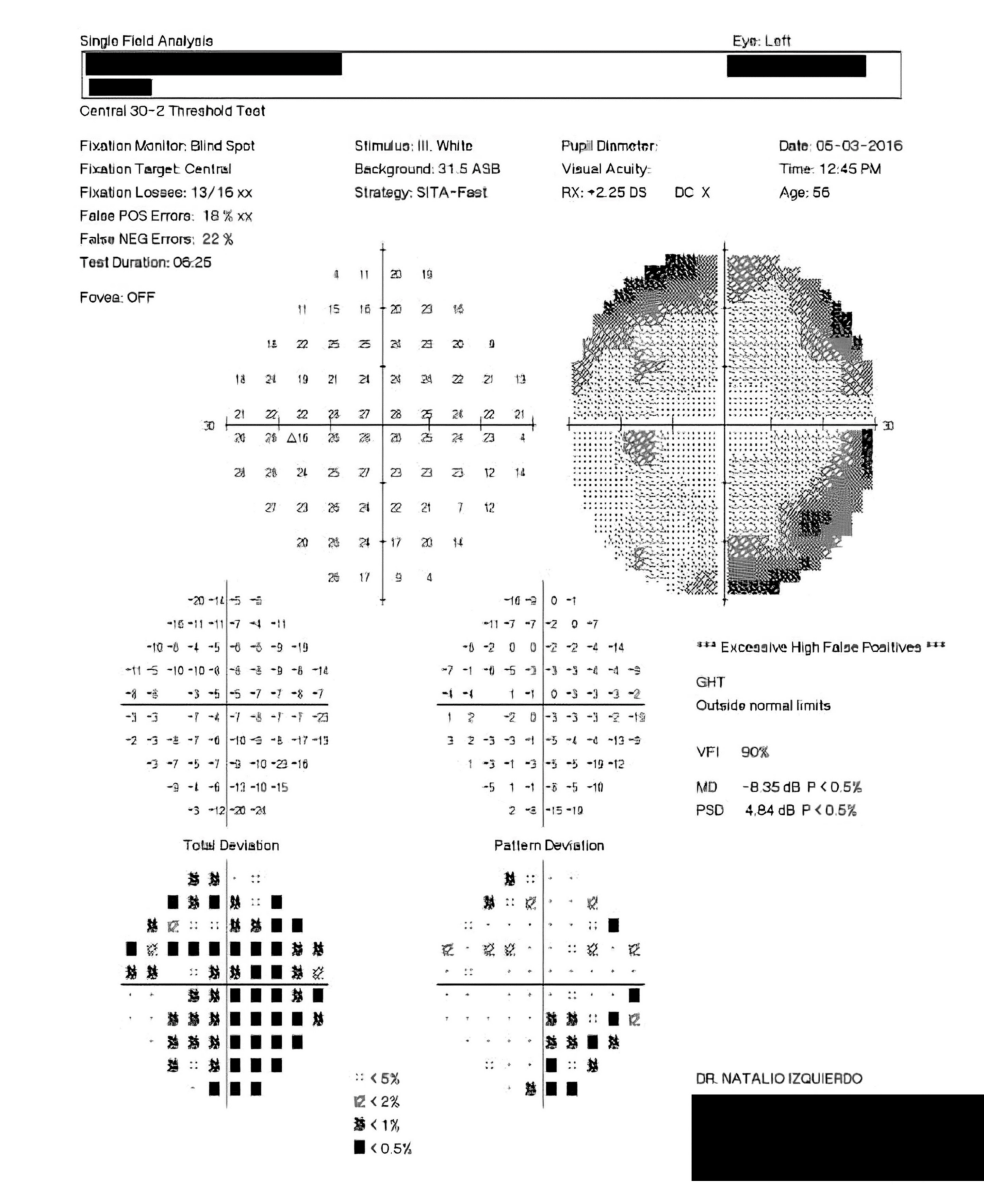
Patient's left eye Humphrey visual field test showing mean deviation of -8.35 dB (30-2*) *Carl Zeiss Meditec AG, Jena, Germany GHT: glaucoma hemifield test; VFI: visual field index; MD: mean deviation; PSD: pattern standard deviation

Upon Optic Nerve Head Analysis done with Stratus Optical Coherence Tomography (OCT) showed a cup-to-disk area ratio of 0.2 and 0.1 in the right and left eye, respectively (Figures 1, 2) despite a Humphrey visual field test being insignificant 1.5 years back. The Humphrey test (30-2; Carl Zeiss Meditec AG, Jena, Germany) showed a mean deviation of -17.27 dB (p<0.5%) and -8.35 dB (p<0.5%), and pattern standard deviation of 10.14 dB (p<0.5%) and 4.84 dB (p<0.5%) in the right and left eye respectively (Figures 3, 4). These findings were not typical of ptosis.

The patient was accompanied by his mother in one of the visits, and she was noted to have ptosis, mild lagophthalmos following surgery, dry eyes, anorexia due to dysphagia, and thus cachexia. The mother was wheelchair-bound due to bilateral lower extremity proximal muscle weakness. This was suggestive of a probable family history.

A clinical diagnosis of OPMD was reached.

However, genetic testing, although planned, could not be done to confirm the clinical diagnosis and there was a lack of detailed medical history of the patient’s mother to compare OMPD timeline presentation and specific symptom onset with the patient. We also could not do any electromyographic studies on the patient to confirm or discard possible subclinical muscle weakness, although through phone calls he has since claimed ambulation limitations due to muscle weakness to explain why he was not been able to come to follow-up appointments.

## Discussion

Previous studies report blepharoptosis as the most common symptom in patients with OPMD [1,3,7]. Our patient had bilateral blepharoptosis at 57 years of age. This finding coincides with prior literature. The second most commonly reported symptom is dysphagia [1,3,7]. The current patient presented with adult-onset dysphagia and exhibited weight loss. This is also consistent with the literature. The third most common symptom is proximal muscle weakness [1,3,7]. The patient did not present with this symptom at the time of diagnosis, which creates a contrast with the standard presentation of OPMD described in previous studies. His mother, however, was wheelchair-bound due to distal muscle weakness.

Patients in the late stages of OPMD may have diverse symptoms due to the specific progression of myopathy. Previous studies report tongue atrophy, dysphonia, limitation of upper gaze, partial horizontal ophthalmoplegia, general facial muscle weakness, which is differentiated from blepharoptosis, loss of tendon reflexes without clear polyneuropathy, and reduced FEV1 [1,3,7]. The current patient did not exhibit any of these symptoms. This could be due to an early diagnosis, epigenetics, or heterogenous phenotype in the patient.

We could not find any other patient with OPMD reported from Puerto Rico, making this case probably the first to be reported from the country.

Limitations of our report include the lack of genetic testing on the patient to confirm the clinical diagnosis, lack of detailed medical history of the patient’s mother to compare OMPD presentation and specific symptom onset with the patient, and lack of electromyographic studies on the patient to confirm or discard possible subclinical muscle weakness.

Further studies should be aimed at assessing the possibility of undiagnosed OPMD patients in rural areas of Puerto Rico, and particularly to this patient, the classification of his late-stage phenotype and presentation with follow-up.

## Conclusions

We presented, classified through symptomatology, and discussed an OPMD patient, who is probably the first reported case from Puerto Rico. Further studies in this area should also include genetic testing or possibly electromyography studies to support the clinical diagnosis. Further studies should be aimed at assessing the possibility of undiagnosed OPMD patients in rural areas of Puerto Rico. We want to elucidate that physicians from Puerto Rico and the Caribbean should not discard these genetic ophthalmologic disorders as differentials due to their rare nature and small patient populations in the area. 
